# Single-site trinuclear copper oxygen clusters in mordenite for selective conversion of methane to methanol

**DOI:** 10.1038/ncomms8546

**Published:** 2015-06-25

**Authors:** Sebastian Grundner, Monica A.C. Markovits, Guanna Li, Moniek Tromp, Evgeny A. Pidko, Emiel J.M. Hensen, Andreas Jentys, Maricruz Sanchez-Sanchez, Johannes A. Lercher

**Affiliations:** 1 Department of Chemistry and Catalysis Research Center, Technische Universität München, Lichtenbergstrasse 4, Garching 85748, Germany; 2 Schuit Institute of Catalysis, Inorganic Materials Chemistry Group, Department of Chemical Engineering and Chemistry, Eindhoven University of Technology, PO Box 513, Eindhoven 5600 MB, The Netherlands; 3 Van't Hoff Institute for Molecular Sciences, University of Amsterdam, PO Box 94215, Amsterdam 1090GE, The Netherlands; 4 Institute for Complex Molecular Systems, Eindhoven University of Technology, PO Box 513, Eindhoven 5600 MB, The Netherlands; 5 Institute for Integrated Catalysis, Pacific Northwest National Laboratory, PO Box 999, Richland, Washington 99352, USA

## Abstract

Copper-exchanged zeolites with mordenite structure mimic the nuclearity and reactivity of active sites in particulate methane monooxygenase, which are enzymes able to selectively oxidize methane to methanol. Here we show that the mordenite micropores provide a perfect confined environment for the highly selective stabilization of trinuclear copper-oxo clusters that exhibit a high reactivity towards activation of carbon–hydrogen bonds in methane and its subsequent transformation to methanol. The similarity with the enzymatic systems is also implied from the similarity of the reversible rearrangements of the trinuclear clusters occurring during the selective transformations of methane along the reaction path towards methanol, in both the enzyme system and copper-exchanged mordenite.

## Abstract (web summary)

Copper-exchanged zeolites with mordenite structure can mimic the active sites in particulate methane monooxygenase. Here, the authors show that mordenite micropores can stabilize trinuclear copper-oxo clusters that exhibit a high reactivity towards activation of carbon–hydrogen bonds in methane.

## Graphical abstract

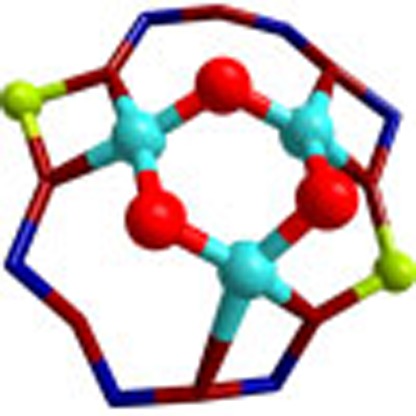

The recent marked increase in the availability of methane as well as its global dispersion requires novel chemistry to convert it into easily condensable energy carriers that can be readily integrated into the existing chemical infrastructure^1^,^2^,^3^. This has triggered a worldwide quest for new processes, allowing economical small-scale operations at remote locations^4^. Such boundary conditions rule out the current dominant method to first convert methane with H_2_O, O_2_ or CO_2_ to synthesis gas (a mixture of CO and H_2_), followed by synthesis of methanol and the subsequent conversion of methanol to hydrocarbons with zeolite catalysts^5^,^6^ or via direct hydrocarbon synthesis via the Fischer–Tropsch process. While oxidative coupling of methane to ethane and ethylene^7^, dehydroaromatization^8^ and the direct partial oxidation of methane to methanol are conceptually promising for a direct conversion of natural gas, only the latter permits lower operating temperatures.

Nature has found a way to convert methane in a single step to methanol via a biocatalytic transformation under aerobic conditions with methane monooxygenase (MMO) as catalysts using Cu and Fe as potential active metals. Two forms of MMOs at different cellular locations are known, a cytoplasmic MMO (soluble MMO) and a membrane-bound MMO (particulate, pMMO). In soluble MMOs, the active site of the hydroxylase contains a bis(μ-oxo)diiron core^9^, while in pMMOs the active site is represented by a Cu cluster that catalyses the insertion of oxygen into the methane C–H bond with a very high rate (rate normalized to the concentration of active sites, that is, the turnover frequency of about 1 s^−1^)^10^.

Aerobic and anaerobic handling during purification led to drastically different concentrations of Cu in the enzyme without inducing marked changes in the enzyme structure, and in turn, differences in the conclusions about the nature of the active site^11^. Rosenzweig *et al.*have attributed the catalytic activity to a Cu dimer^12^,^13^,^14^, while Chan *et al.* suggested a cluster of three Cu atoms to form the active site^11^,^15^,^16^,^17^. Following these leads, homogeneous^18^,^19^,^20^ and heterogeneous^21^,^22^ Cu-based catalysts have been explored. In particular, studies of the heterogeneous Cu catalysts led to limited and conflicting insights despite the excellent spectroscopic work, because in nearly all cases the active site has been concluded to represent only a minority of the Cu species in the catalyst.

Using leads, especially from the work on zeolite catalysis for the conversion of methane to methanol^23^,^24^,^25^,^26^,^27^,^28^, we develop an approach to prepare uniform Cu-oxo species in zeolites able to activate and convert methane. Here, we demonstrate that by using this approach, a selective stabilization of single-site Cu-oxo clusters in mordenite micropores is possible. Zeolite mordenite (MOR) has non-intersecting 12-membered ring (12-MR) channels with 8-membered ring (8-MR) pockets. In mordenite, not only the uniformity of Cu species but also a high concentration of these sites is achieved, resulting in an unprecedented activity of the material. During methane oxidation, these sites dynamically disintegrate and reform upon reoxidation, a rearrangement that is needed to close the (catalytic) cycle.

## Results

### Formation of an active copper single site in mordenite

**Figure 1:**
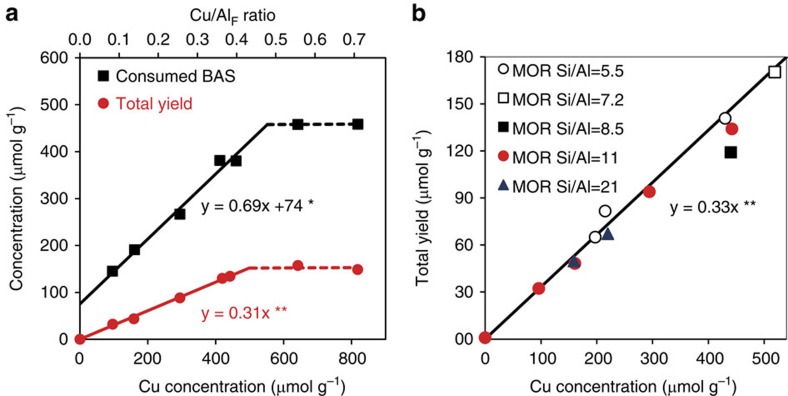
Activity and framework aluminium coordination upon copper loading. (**a**) The concentration of tetrahedrally coordinated aluminium acting as an ion exchange site for Cu^2+^ with total yield for Cu-MOR with Si/Al=11 and (**b**) total yield of methane oxidation as a function of Cu concentration in Cu-MOR for various Si/Al ratios. *The slope of 0.69 indicates an exchange stoichiometry of 2/3 meaning that two H^+^ are substituted by three Cu^2+^. The offset of 74 μmol g^−1^ shows slight dealumination of framework Al (∼5%) during Cu exchange. **The slopes of 0.31 and 0.33, respectively, indicate that three Cu centres are involved in the oxidation of one methane molecule.

For preparation of a single type of cluster on a solid support via ion exchange sites, two main requirements have to be met, that is, (i) the exchanging species must be well defined in the aqueous solution, and precipitation of the metal cations by changes in the pH during the exchange procedure has to be avoided; (ii) the exchanged cations need to form a well-defined thermodynamically stable configuration upon coordination to the zeolite lattice. We have selected mordenite as a matrix for the synthesis of Cu clusters, because it is known that this framework allows a preferential exchange of the sites located in the more constrained side pockets (SP)^29^. The ion exchange was carried out using Cu-acetate as a precursor and under conditions (pH=5.7) to maximize the concentration of partially hydrolysed Cu(OH)^+^ ions to avoid their further hydrolysis and precipitation as Cu(OH)_2_. The concentration of alkali cations that could compete for the exchange sites in the zeolite support was also minimized. Using this approach, a series of ion-exchanged Cu-MOR catalyst precursors with varying Cu concentrations were prepared. Subsequent calcination in flowing O_2_ at 450 °C converted these precursors to active materials. The high-temperature activation is necessary for the dehydration of the materials and the induced migration of the exchanged Cu ions towards formation of the oxide clusters. In the oxidized and dry state, it was surprisingly observed that only two lattice aluminium ions were involved in binding of three Cu cations (Fig. 1a). A blank experiment showed the preparation procedure eliminates ∼5% of the total Brønsted acid sites (BASs), ca. 70 μmol g^−1^, by dealumination.

The activity of these clusters was evaluated by exposing the activated catalyst to methane at 200 °C followed by purging the zeolite with water to release the formed products. Approximately 80% of the methane converted by the materials was desorbed as methanol or dimethyl ether in the purge step. The total yield of methane oxidation products per gram of zeolite catalyst was an order of magnitude higher than the maximum methanol yields reported in the recent literature for this catalyst class (160 versus 13 μmol g^−1^)^27^,^30^. The productivity of the active materials scaled linearly with the Cu concentration pointing to a stoichiometry of three Cu cations needed to convert one methane molecule (Fig. 1b). The linear dependence of the activity on the Cu^2+^ concentration strongly suggests that only one type of active site has been formed on a large series of samples with different Cu loading. The stoichiometry of methane activated per Cu, together with the observed Cu/consumed BAS ratio valid for different Si/Al ratios (see Supplementary Fig. 1 and Supplementary Table 1) is the first evidence of an active site involving three Cu atoms anchored to two Al framework sites. With these findings in hand, we redirected our attention to the nature of these sites and the origin of their high reactivity towards methane.

### Siting of copper-oxo species

The first key question to be addressed is the structure and location of the active Cu-oxo cluster within the zeolite micropores. The use of different probe molecules has allowed a precise determination of the acid site distribution in H-MOR, which can be controlled by varying the zeolite synthesis conditions. The diameter of MOR side pockets (SP,8-MR) is substantially smaller than the aperture of the large straight channels composed of 12 Si(Al) atoms. Pyridine, owing to its basicity and spherical shape, can access both BASs located in the main channel, as well as in the pore mouth of the side pockets^31^. On the contrary, the more bulky elongated *n*-hexane molecule interacts only with BASs in the large 12-MR channel^32^. By analysing the results of infrared spectra of pyridine and *n*-hexane adsorption, it was demonstrated that the fraction of BASs in MOR side pockets, which are inaccessible for *n*-hexane, constitute about 65% in the H-MOR used in this study (see Supplementary Fig. 2). From this amount, 55% of BASs are accessible to pyridine, meaning that these protons are in the side pockets but located close to the 8-MR mouth pore.

To obtain insight into the location of extra-framework Cu clusters, we employed the same approach to investigate the distribution of residual BAS in Cu-MOR materials after their activation in O_2_. By direct comparison of the concentration of OH groups associated with BAS in the activated Cu-MOR with the concentration of these groups of the parent H-MOR, the location of the Cu-oxo clusters was inferred. It should be emphasized that deconvolution of the SiO-HAl vibration band associated with BAS did not show a change of concentration of BAS in the main channel (3,612 cm^−1^) with Cu loading. Only the band associated with BAS in the side pockets (3,590 cm^−1^) decreased with increasing Cu concentration. Perturbation of the main channel BAS with *n*-hexane confirmed these findings. In turn, this allows the conclusion that Cu^2+^ exchanges selectively for H^+^ in the side pockets. It is hypothesized that the relatively high concentration of framework Al (65% of the total) in the side pockets is stabilizing the Cu-oxo ions. A linear decrease of BAS concentration probed by pyridine adsorption with increasing Cu loading indicates location of the Cu ions balancing Al atoms near the pore mouth of the MOR side pocket (see Table 1 and Supplementary Fig. 3).

In addition, the concentration of Al sites paired in the MOR samples was probed by Co^2+^ exchange, following the method described in ref. 33. It was observed that a majority of Al atoms (66% for Si/Al 11; 60% for Si/Al 21) are separated from another by only one or two Si units. Combining this information, it is concluded that the Cu clusters are balancing the charge of two Al sites located in the 8-MR of the side pockets.

### Structure determination of the copper-oxo active site

**Figure 2:**
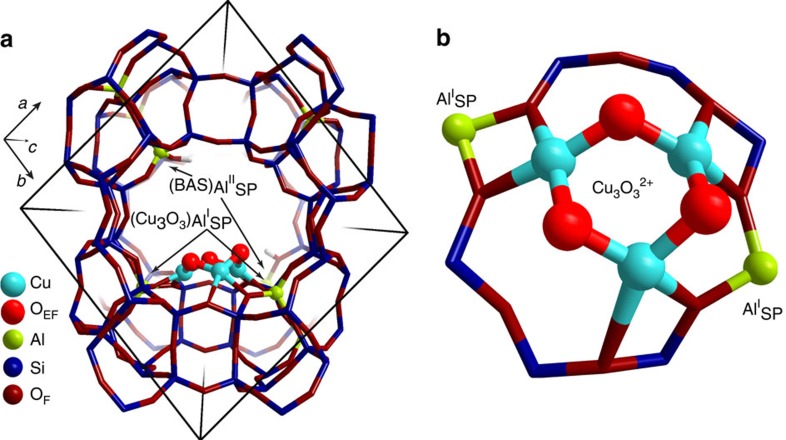
Structure and location of [Cu_3_(μ-O)_3_]^2+^ cluster in mordenite predicted by DFT. The zeolite model contained paired (type I) and isolated (type II) Al atoms located at the pore mouth of the side pocket. The cluster is stabilized by two anionic centres due to Al^I^_SP_ lattice sites at the entrance of the MOR side pocket (**b**) so that the extra-framework oxygens responsible for the initial C–H activation are pointing towards the main channel of MOR (**a**). The charge due to the remaining Al^II^_SP_ is compensated by acidic protons resulting in BAS formation.

The information obtained about the nuclearity and location of the Cu-oxo active sites was used to propose several model structures for trinuclear Cu clusters in MOR to be studied by density functional theory (DFT) calculations. Their stability was evaluated and compared with dicopper clusters, as those are typically described to be the active species for Cu-ZSM-5 in the literature^34^. Calculated reaction energies for interconversion of different Cu species indicated a high intrinsic stability of binuclear complexes at 0 K. However, *ab initio* thermodynamic analysis in terms of reaction Gibbs free energy depending on system temperature and pressure showed that the [Cu_3_(μ-O)_3_]^2+^ complex (Fig. 2) is the most stable species at 700 K in O_2_ atmosphere and under dry conditions (see Supplementary Fig. 4). The proposed stoichiometry suggests a formal mixed Cu(II)/Cu(III) composition of the cluster. However, analysis of the electronic properties of the computed structures indicates that, because of the substantial anion-radical nature of the oxygen ligands (Bader charge −0.76 *e* and −0.63 *e* compared with the Bader charge of −1.09 *e* on oxygens in bulk CuO), all Cu sites in the trinuclear cluster are more adequately described as being Cu(II) because their charges are very close to those computed for Cu centres in bulk CuO (+1.09 *e*). The results of Bader charge and spin-polarized charge density analysis are summarized in Supplementary Fig. 5.

To verify the nature of the Cu-oxo cluster predicted by DFT calculations, we analysed the Cu species of an activated Cu-MOR sample by X-ray absorption spectroscopy (XAS). To date, XAS analysis has not provided unambiguous information on the nature of the active Cu species in Cu-zeolites, due to the heterogeneity of the Cu species in conventional materials. However, the reactivity data discussed above point to a uniform nature of the Cu sites in the Cu-MOR materials prepared by the optimized procedure presented here and, therefore, Cu K-edge spectra may yield direct information of the structure of the active clusters.

**Figure 3:**
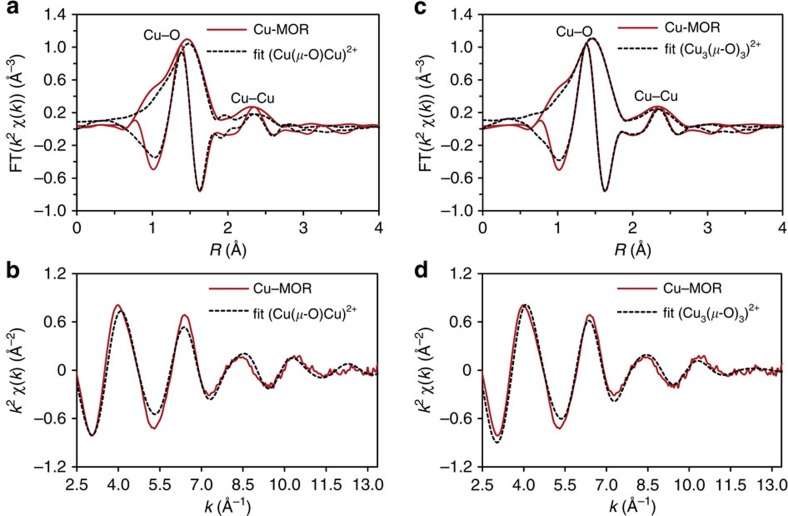
Copper EXAFS data and fitting for Cu-MOR. Comparison of the *k*^2^-weighted Fourier transformed EXAFS at the Cu K-edge of the Cu-MOR zeolite activated in O_2_ at 450 °C with EXAFS simulation of an intrazeolite (**a**) binuclear [Cu(μ-O)Cu]^2+^, (**c**) trinuclear [Cu_3_(μ-O)_3_]^2+^ complexes and (**b**,**d**) the corresponding *k*^2^-weighted experimental EXAFS oscillations and their simulation using the DFT-computed model. Colour key: measured spectra (red lines), simulated spectra (black lines).

Figure 3a,b compares the *k*^2^-weighted and Fourier transformed EXAFS (Extended X-ray Absorption Fine Structure) data measured at the Cu K-edge with the simulated EXAFS, using the DFT-optimized [Cu(μ-O)Cu]^2+^ cluster in the MOR unit cell with a structure previously proposed as the active site for methane activation in Cu-ZSM-5 (ref. 35). The multiple k-weighted analysis is crucial for a reliable analysis to recognize and properly analyse both light and heavy scatterers and, in addition, to consider the anti-phase behaviour of the different Cu–Cu and Cu–O shells, with constructive and destructive interferences in difference parts of the EXAFS range^36^. Therefore, the full EXAFS data were analysed in k- and R-space using a combined *k*^1^–*k*^2^–*k*^3^-fitting procedure. A fit of the fully refined cluster was only acceptable, if the fit was of high quality in all k-weightings.

The most prominent peak can be observed below 2 Å in *R*-space and is associated with backscattering from the oxygen atoms to which Cu is directly bonded. The complex line shape suggests at least two distinct types of O-donor ligands with significantly different Cu–O distances due to framework (O_F_) and extra-framework oxygen (O_EF_). Features above 2.0 Å in *R*-space arise from Cu–Cu and second shell Cu–O single-scattering paths. The experimental data significantly deviates from the simulated spectrum of a binuclear [Cu(μ-O)Cu]^2+^ complex. A particularly strong deviation can be seen at large interatomic distances (*R*∼2.25 Å—not phase corrected), corresponding to the second coordination shell of Cu. Multiple scattering paths must be visible for a dimeric structure with a O–Cu–O structure, similar to typical CuO spectra^37^. The absence of this feature in the experimental spectra suggests the existence of >1 Cu–Cu path and, therefore, the presence of a Cu cluster with a nuclearity higher than 2. Conversely, a good fit of the experimental EXAFS data is achieved by the simulated EXAFS based on the DFT-optimized structure of the [Cu_3_(μ-O)_3_]^2+^/MOR cluster model (Fig. 3c,d and Supplementary Fig. 6 for comparison of *k*^1^, *k*^2^ and *k*^3^ weighted plots). Results of the fitting are summarized in Table 2. The DFT-optimized geometric parameters of the proposed trinuclear Cu cluster show a low symmetry of this species (see Supplementary Table 2). Therefore, Cu atoms in the cluster are not equivalent and two different shells need to be included. Coordination numbers (CNs) are averaged for the three Cu scatterers. The average CNs derived from the DFT-optimized geometric parameters of the most stable trinuclear cluster are applied for the fit to take all Cu–O and Cu–Cu contributions into account (see Supplementary Tables 2 and 3). When the EXAFS fitting was started from an alternative trinuclear model, similar parameters were obtained. This indicates that the values shown in Table 2 are a true minimum. Details on the EXAFS fitting for different model clusters are available in Supplementary Tables 4 and 5.

### *In situ* monitoring of active copper species

**Figure 4:**
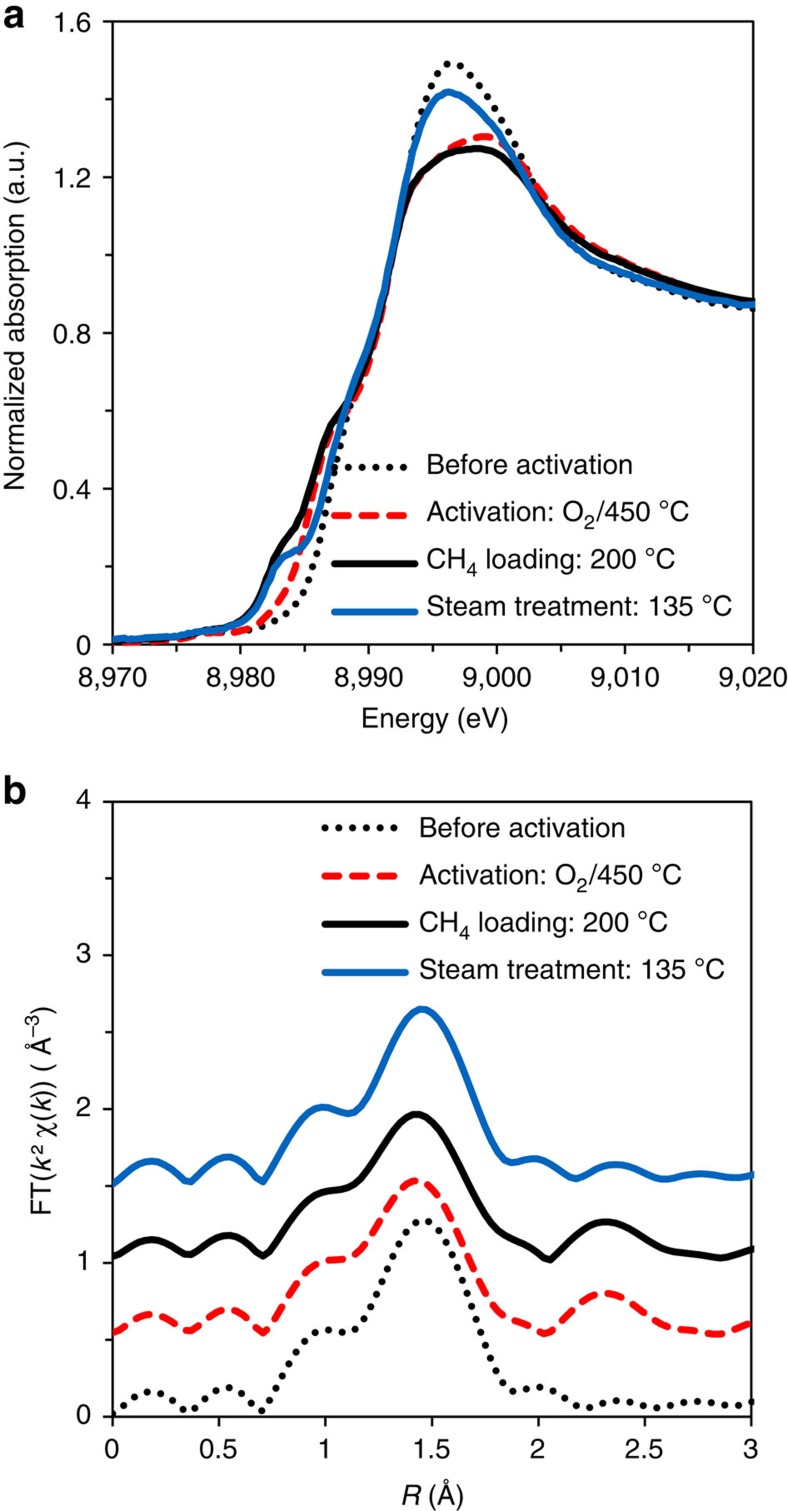
*In situ* X-ray absorption spectroscopy. (**a**) *In situ* XANES and (**b**) Fourier transformed EXAFS during a full cycle of selective partial oxidation of methane to methanol.

Having shown that the single site in the present Cu-MOR catalysts is a [Cu_3_(μ-O)_3_]^2+^ cluster, the next step was to monitor the formation of the cluster during activation under O_2_ and its interaction with CH_4_ under reaction conditions. For this purpose, an *in situ* study was performed by XAS and ultraviolet–visible (UV-vis) spectroscopy. Figure 4 shows the XANES (X-ray Absorption Near Edge Structure) and EXAFS of Cu-MOR at different stages of the catalytic cycle. Dehydration of the fresh catalysts led to a change from the octahedral coordination sphere Cu^2+^ in hexaquo-complexes to tetrahedral Cu^2+^ species. At temperatures above 200 °C, the progressive formation of a feature at *R*>2 Å in EXAFS was observed (see Supplementary Fig. 7), which can be attributed to the new Cu–Cu path and therefore to the formation of Cu-oxo clusters with nuclearity ≥2.

*In situ* ultraviolet–visible spectroscopy of the activation of Cu-MOR in O_2_ showed the development of a very broad band centred at ca. 31,000 cm^−1^ (see Supplementary Fig. 8), while a band at 22,700 cm^−1^, assigned to extra-framework O→Cu(II) charge transfer for the active species [Cu(μ-O)Cu]^2+^ in Cu-ZSM-5 (ref. 35), was not observed in any of the tested conditions. This fact further supports the conclusion that the active Cu-oxo clusters reported here have a structure different from those described for conventionally prepared Cu-ZSM-5.

**Figure 5:**
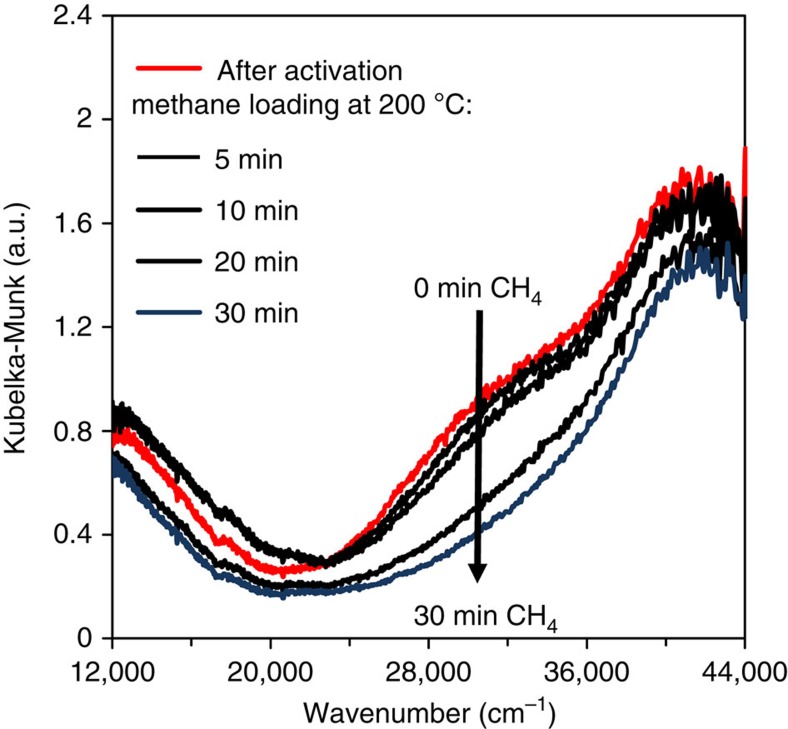
Ultraviolet–visible spectroscopy of Cu-MOR. *In situ* UV–vis spectra of Cu-MOR after activation in oxygen at 450 °C and subsequent methane loading at 200 °C.

Upon reaction with CH_4_, the development of a new strong feature at 8,983 eV has been observed in XANES, which indicates a reduction of a fraction of intrazeolite Cu^2+^ to Cu^+^ (Fig. 4a). This is in line with the thermal autoreduction reported in the literature^37^,^38^, and also in good agreement with different mechanisms proposed for the donation of an oxygen atom from the metal oxide cluster to the methyl moiety^39^. On the other hand, significant changes could not be noted in the Fourier transformed EXAFS upon the treatment with CH_4_, indicating that the trinuclear structure of the active site is preserved at this step, presumably because the oxygenated products remained strongly attached to the cluster. In the UV–vis spectra, the broad band centred at 31,000 cm^−1^, which is stable at 200 °C under O_2_ or N_2_, disappears only after 20–30 min contact with CH_4_ flow at 200 °C (Fig. 5). The low rate of disappearance of this feature is in good agreement with the low rate of reaction predicted for the catalyst (at least 30 min in contact with CH_4_ was necessary to measure significant amounts of methanol).

### Mechanism of C–H bond activation on [Cu_3_(μ-O)_3_]^2+^ cluster

To understand the elementary energetics of methane to methanol oxidation on the proposed trinuclear complex [Cu_3_(μ-O)_3_]^2+^ on a more quantitative mechanistic basis, DFT calculations were performed. The methane activation proceeds via a homolytic C–H bond cleavage followed by a direct radical rebound reaction mechanism^34^, resulting in methanol adsorbed to a reduced Cu cluster. The C–H bond activation over the extra-framework Cu_3_O_3_^2+^ cluster is facilitated by the interaction with a formally radical-anionic extra-framework oxygen centre. The interaction of the respective single-occupied molecular orbital of Cu_3_O_3_^2+^ with the antibonding CH orbital (σ*(CH)) of methane results in the cleavage of the CH bond, resulting in CH_3_^·^ and an OH group bound to the cluster (see Supplementary Fig. 9). The role of Cu centres and, accordingly, the orbital interactions with the metal ions, is to stabilize the electronic configuration of the extra-framework cluster with the anion-radical character of the oxygen ligands necessary for the facile C–H activation^40^. The C–H bond activation barrier of 74 kJ mol^−1^ (see Supplementary Fig. 10) is comparable to that of 78 kJ mol^−1^ on binuclear [Cu(μ-O)Cu]^2+^ in ZSM-5 (ref. 34). The CH activation is followed by a barrierless recombination of the CH_3_ radical with the Cu-bound OH group in the cluster. The so-formed methanol molecule is preferentially coordinated to two neighbouring Cu centres of the trinuclear species (see Supplementary Fig. 10), in good agreement with the conservation of the cluster structure upon methane activation observed by XAS.

As has been outlined above, desorption of methanol is only accomplished by steam treatment of the catalysts at 135 °C, which led to a substantial decrease of the Cu–Cu path in the EXAFS (Fig. 4b). We conclude from these data that the trinuclear Cu cluster is hydrolysed by contact with water. Interestingly, the renewed activation of the material in O_2_ at 500 °C completely restored the activity, even when the procedure was repeated up to eight cycles (see Supplementary Fig. 11). Identical XAS and UV–vis spectra were obtained for samples after a second activation, confirming that the [Cu_3_(μ-O)_3_]^2+^ cluster is highly stable and re-forms by self-organization under dry oxidation conditions in mordenite.

## Discussion

The choice of a MOR with high concentration of Al in the side pockets, together with an optimized synthetic approach for copper exchange has yielded Cu-MOR materials, showing an outstanding activity in methane activation, which is at least one order of magnitude higher than those reported in the literature for analogous systems. In contrast to other metal-exchanged zeolites where a mixture of cationic species with different structures and reactivities is detected^41^,^42^, the stoichiometry of converted methane to Cu for these Cu-MOR materials has shown that it is possible to develop a Cu zeolite with only one type of active site.

*In situ* XAS demonstrated that the homogeneous single sites in activated Cu-MOR are the trinuclear Cu-oxo clusters, namely [Cu_3_(μ-O)_3_]^2+^, anchored to two framework Al atoms located at the pore mouth of the 8-MR side pockets. Furthermore, this active [Cu_3_(μ-O)_3_]^2+^ species has been found to be highly stable under dry conditions, in agreement with *ab initio* thermodynamic analysis based on DFT results. Even though the reaction with methane followed by steam treatment led to the hydrolysis of the cluster, it can be re-formed without loss of activity by reactivation in O_2_.

The present results show conclusively that trimeric Cu-oxo clusters are active and selective for partial methane oxidation. It should be noted in this context that Chan *et al.*, recently proposed such a cluster to be active in pMMO and reported that it was possible to use tri-copper complexes for selective methane oxidation to methanol, albeit with H_2_O_2_ as an oxidant^17^,^43^. The multiple Al framework atoms in the 8-MR side pockets of H-MOR provide the conditions to stabilize [Cu_3_(μ-O)_3_]^2+^ clusters. We hypothesize, in addition, that the 8-MR side pockets in MOR enhance the activity of the clusters by providing similar steric constraints as found for the hydrophobic cavity formed by the pmoA and pmoC subunits of pMMO^15^,^16^.

The material presented here is one of the few examples of catalysts with well-defined active sites evenly distributed in the zeolite framework, a truly single-site heterogeneous catalyst. This not only allows for much higher efficiencies in conversion of methane to methanol than previously reported, it also enables the unequivocal linking of the structure of the sites with their catalytic activity. Understanding why Cu clusters form reversibly, in varying reaction environments, while similar clusters remain stable in the enzyme, despite unfavourable conditions, is one of the big hurdles to achieve similar activities and selectivities in heterogeneous catalysts, as are usually found in enzymatic systems alone. The presented system is therefore a more than promising basis to tackle this challenge.

## Methods

### Preparation of Cu-exchanged zeolites

H-MOR was obtained by calcination of commercial zeolite NH_4_-MOR (Clariant, Si/Al 11, 21) in synthetic air at 500 °C for 8 h. Cu-MOR with different Cu/Al ratios was prepared by aqueous ion exchange of H-MOR with Cu^2+^. The Cu^2+^ exchange was carried out at ambient temperature by contacting 5 g zeolite with 300 ml of aqueous Cu(CH_3_COO)_2_ (Sigma-Aldrich, 99.99%) solution. The reaction time and the molarity of the solution was varied between 0.0025 and 0.01 M Cu(CH_3_COO)_2_ to obtain Cu/Al ratios between 0.1 and 0.4. A series of several subsequent cycles of ion exchange with intermediate rinsing was performed in an attempt to increase the Cu/Al ratio (0.4–0.6). The pH of the solution was 5.5–6.0 during exchange. A typical exchange time was 20 h. After the last exchange step, the samples were rinsed four times with doubly deionized water (50 ml g^−1^ MOR each time) with an intervening centrifugation step between each rinse. These rinse cycles were performed to ensure that the pores did not contain further non-exchanged Cu ions, which would form large CuO clusters during activation. Samples were then dried in static ambient air at 110 °C for 24 h. The Si, Al, Na and Cu contents were measured by atomic absorption spectroscopy on a UNICAM 939 AA spectrometer after dissolution in boiling hydrofluoric acid. Brunnauer-Emmet-Teller (BET) surface area was measured on a PMI automated Sorptomatic 1990 after activation at 350 °C. Co^2+^ exchange was prepared by aqueous ion exchange of Na-MOR in 0.05 M Cu(NO_3_)_2_ solution at room temperature, following the procedure described in ref. 33. Na-MOR was prepared by Na^+^ exchange of freshly calcined H-MOR with 0.5 M NaNO_3_ solution for 24 h at 60 °C.

### Testing of activity for selective oxidation of methane

Cu-MOR samples were tested for their activity towards methane oxidation in an atmospheric pressure stainless steel plug flow reactor with a 4-mm inner diameter. The reaction included three consecutive steps: (i) activation, (ii) CH_4_ loading and (iii) steam-assisted CH_3_OH desorption. In a typical experiment, 0.1 g of Cu-MOR (250–400 μm) was calcined in an O_2_ flow (16 ml min^−1^) at 450 °C for 1 h. The activated catalyst was cooled to 200 °C in O_2_ and flushed in He. In the subsequent CH_4_-loading step, 90% CH_4_ in He (16 ml min^−1^) was passed over the sample for 4 h. The temperature was then decreased in He to 135 °C. A steam-assisted CH_3_OH desorption step was carried out by passing an equimolar mixture of H_2_O steam and He (20 ml min^−1^) through the reactor bed for 30 min. The reaction products were identified and quantified by online mass spectroscopy by monitoring the time-dependent evolution of signals at *m/e* 28, 31, 44 and 46 characteristic for CO, CH_3_OH, CO_2_ and (CH_3_)_2_O, respectively. The He signal (*m*/*e*=4) was used as an internal standard. Productivity was calculated as the product of the effluent flow rate and the integral of the product concentrations as a function of time. The product (CH_3_)_2_O was assumed to be formed via condensation of two partially oxidized CH_4_ molecules corresponding to two CH_3_OH equivalents. The sum of all detected products is referred to as total yield.

### Infrared spectroscopy

The samples for infrared spectroscopy were prepared as self-supporting wafers with a density of ca. 10 mg cm^−2^. Samples were first activated in vacuum (1.0 × 10^–7^ mbar) at 450 °C for 1 h with a heating rate of 10 °C min^−1^. Infrared spectra of adsorbed *n*-hexane were recorded on a Vertex 70 spectrometer from Bruker Optics at a resolution of 4 cm^–1^. After pretreatment, the activated samples were cooled to 30 °C, *n*-hexane (0.5–5 mbar) was adsorbed and equilibrated for at least 30 min. All spectra were recorded at 30 °C. Infrared spectra of adsorbed pyridine were measured on Thermo Nicolet 5,700 FT-IR spectrometer with a resolution of 4 cm^−1^. After activation, the total concentration of BAS was determined at 150 °C after adsorption of 0.1 mbar pyridine and subsequent evacuation for 30 min at the same temperature. All spectra were recorded at 150 °C.

### DFT calculation

All periodic DFT calculations were performed using VASP software with a generalized gradient-approximated PBE exchange-correlation functional^44^,^45^. Projected augmented wave method and plane wave basis set with a cutoff of 400 eV were employed. Brillouin zone-sampling was restricted to the *Γ* point^46^. A supercell of all-silica MOR, constructed by a doubling monoclinic primitive cell along the *c* axis with lattice parameters of *a*=*b*=13.648, *c*=15.015 Å and γ=97.2° as optimized by DFT, was used as an initial model^47^. To compensate for the positive charge of the extra-framework cationic Cu complexes, two framework Si^4+^ ions in the MOR supercell were substituted by two Al^3+^ at the side-pocket position of Al^I^_SP_. The other two [AlO_2_]^−^ units at the side-pocket position of Al^II^_SP_ were charge compensated by two BASs^48^. The resulting MOR model had a Si/Al ratio of 11. The nudged elastic band method^49^ was used to determine the minimum energy path and to locate the transition-state structure for the methane oxidation to methanol reaction. The maximum energy geometry along the reaction path obtained by the nudged elastic band method was further optimized using a quasi-Newton algorithm. In this step, only the extra-framework atoms were relaxed. Spin-polarized calculations were performed throughout this study. The calculated reaction paths following the spin PESs (potential energy surfaces) of ground electronic states with *S*=1/2 and *S*=3/2 were very close in energy both for intermediates and transition states of methane activation. Vibrational frequencies were calculated using the finite-difference method, as implemented in VASP. Small displacements (0.02 Å) were used to estimate the numerical Hessian matrix. The transition state was confirmed by the presence of a single imaginary frequency corresponding to the reaction path. Electron density analysis was carried out using VESTA^50^.

For molecular orbital analysis, single-point calculations at the PBE/6-31+G(d,p) level of theory were carried out using the Gaussian 09 program^51^ on a 8-MR cluster model (see Supplementary Fig. 9) directly cut from the periodic DFT-optimized Cu_3_O_3_/MOR structure. The dangling Si–O bonds at the periphery of the cluster model were saturated by hydrogen atoms oriented in the direction of the next T-sites of the zeolite lattice. Both doublet and quartet ground spin states (*S*=1/2 and *S*=3/2) were considered.

### *Ab initio* thermodynamic analysis

To account for the effect of temperature as well as the presence of H_2_O and O_2_ upon the catalysts activation on the stability of different extra-framework Cu complexes in Cu/MOR, *ab initio* thermodynamic analysis was employed. In this study, the thermodynamic analysis is performed with a reference to bulk copper oxide as the most plausible alternative to extra-framework Cu species formed in the zeolite. The following reversible reactions were considered to compare equilibria among species with different chemical compositions:


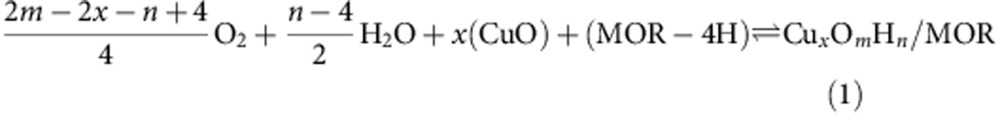


The reaction Gibbs free energy *ΔG* for equilibrium (1) is:


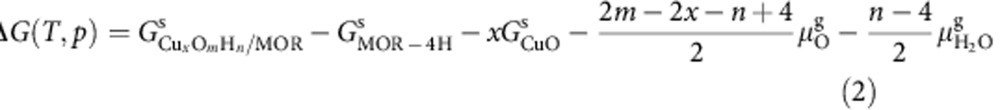


The vibrational and pressure-volume contributions of solids are neglected, and the Gibbs free energies of zeolite and bulk copper oxide are approximated as their respective electronic energies directly computed by DFT. The chemical potentials of the gas phase O (O_2_) and H_2_O depend on *T* and *p*. We assume that the surrounding O_2_ atmosphere forms an ideal gas-like reservoir, and we chose the reference state of 
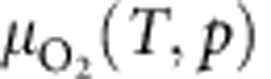
 to be the total electronic energy of an isolated O_2_ molecule (
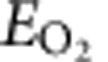
). In other words, the chemical potential of oxygen at the reference state at 0 K is 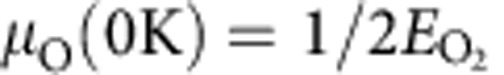 in which 
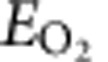
 is the DFT-calculated total energy of O_2_. Then the chemical potential of oxygen at arbitrary *T* and *p* can be written as:


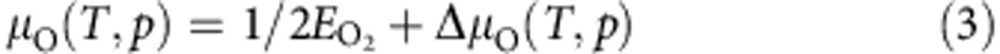


Where


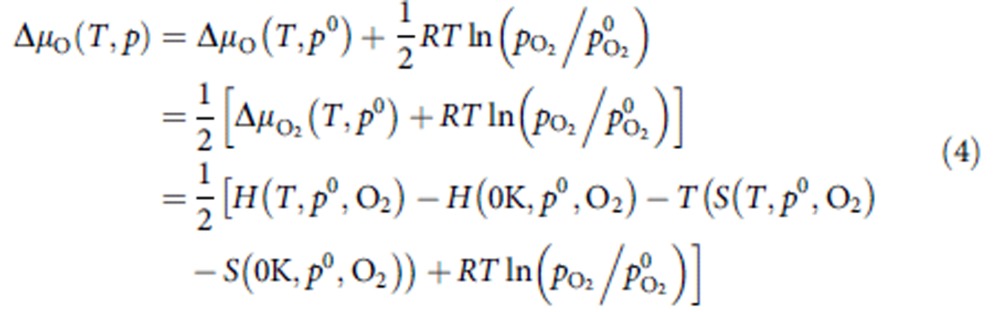


The chemical potential change (Δμ_O_(*T*,*p*)) defined in such a manner includes all temperature- and pressure-dependent free-energy contributions. The temperature and pressure dependency of the chemical potential is obtained from the differences in the enthalpy and entropy of an O_2_, as well as H_2_O molecules with respect to the reference state at the 0-K limit. For standard pressure (1 atm), the values tabulated in thermodynamic tables were employed^52^. This approach has been previously proven to provide rather accurate results for *ab initio* thermodynamic calculations^53^,^54^.

The chemical potential of H_2_O as well as the chemical potential change were calculated exactly in the same way as described in the above:


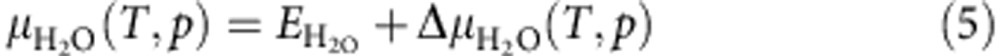


Bringing equations (3) and (5) into equation (2) and considering the Gibbs free energies of zeolite and bulk copper oxide are approximated as their respective DFT-computed electronic energies, we arrive at:


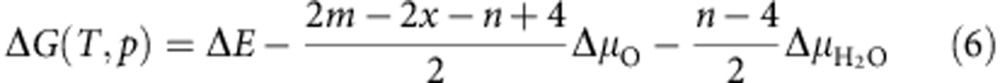


where


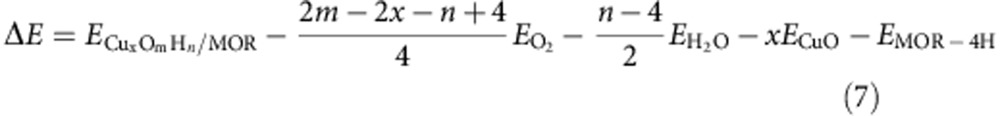



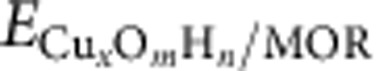
 is the total electronic energy of a given Cu-containing MOR model, *E*_MOR–4H_ is the energy of the H-form of MOR with four Al^3+^ substituted at Al^I^_SP_ and Al^II^_SP_, *E*_CuO_, 
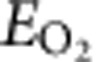
 and 
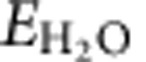
 correspond to the electronic energies of bulk CuO, gaseous H_2_O and O_2_, respectively. The factor *x* denotes the number of Cu atoms in the unit cell of Cu_*x*_O_*m*_H_*n*_/MOR. Depending on the structure of the Cu complex, *x* can be 1, 2 or 3.

### X-ray absorption spectroscopy

X-ray absorption spectra were recorded at Diamond Light Source in Oxford Shire, UK, on beamline B18. The electron energy was 3 GeV with a beam current of 300 mA. The beam size at the sample was 200 × 250 μm. Samples were prepared as self-supporting wafers (60–80 mg) and placed into an *in situ* XAS cell. The X-ray absorption spectra were collected *in situ* at the Cu K-edge (8,979 eV) during activation in oxygen at 450 °C, during exposure of the sample to CH_4_ and after steam treatment. To avoid condensation, all lines of the set-up were thermostated at 110 °C. The samples were activated in an O_2_ flow of 30 ml min^−1^ at 450 °C for 1 h (heating ramp 10 °C min^−1^) and afterwards cooled to 200 °C. After a short flush with He, CH_4_ was loaded for 4 h at 220 °C (flow 30 ml min^−1^). The temperature was then decreased under He flow to 135 °C and an equal molar mixture of water steam/He (50 ml min^−1^) was passed for 2 h through the cell. The Cu K-edge XANES data processing and EXAFS analysis were performed using IFEFFIT version 1.2.11d with the Horae package (Athena and Artemis)^55^,^56^. The amplitude reduction factor, that is, *S*_0_^2^, was experimentally derived to be 0.9, from EXAFS analysis of Cu reference compounds with known structures, that is, Cu(OAc)_2_ and Cu(OH)_2_ (refs 57, 58). Fitting was done in k- and R-space and in multiple weightings of *k*^1^, *k*^2^ and *k*^3^, simultaneously. A fit was only concluded to be good, if all fits in all weightings, as well in k- as in R-space were good and all included contributions were determined to be significant, tested by refinement of CNs. Refinement of CNs gave values with a deviation of <10% from the values predicted by the DFT model for all refined paths. Fits were performed using the optimized geometrical parameters for Cu-MOR obtained from the periodic DFT calculations as an input model.

### EXAFS fitting and analysis

For the fitting of EXAFS spectra, the amplitude (*S*_0_^2^), determined from reference materials, as well as the CNs derived from the DFT models were fixed to reduce the number of fitting parameters. In a second step, Debye–Waller factors were fixed to the values obtained in the best fit with set CNs, and thus CNs and bond distances were refined. Only if the refined values of the distances and the corresponding CNs were in good agreement with the DFT model, a fit was considered as good. Although the *R*-factor (0.009, see Supplementary Table 4) is low, a significant deviation between the experimental data and the DFT model of a binuclear [Cu(μ-O)Cu]^2+^ complex can be observed and is particularly pronounced at larger interatomic distances (*R*) (see Fig. 3a,b). Not just the intensity of the fitted model, but also the imaginary part of the Fourier transform, does not fit well in the Cu–Cu region. Moreover, large Debye–Waller factors with large statistical errors are obtained for both the Cu–Cu and Cu–O_F_ contributions (Supplementary Tables 4 and 5). Possible multiple scattering paths, likely for a dimeric structure with fairly linear O–Cu–O structure motifs and, for example, clearly pronounced in CuO^37^, cannot be observed or analysed. This suggests the presence of >1 Cu–Cu path and therefore the presence of a Cu species with nuclearity higher than 2. Comparison of various Cu trinuclear models proposed by DFT (Supplementary Fig. 4) showed that a good fit of the experimental EXAFS data is achieved by a trinuclear [Cu_3_(μ-O)_3_]^2+^ cluster (Fig. 3c,d), in good agreement with our calculations predicting this cluster to be the most stable under activation conditions. Results of the fitting are summarized in Table 2. It should be noted that the three Cu atoms in the trinuclear [Cu_3_(μ-O)_3_]^2+^ cluster have the same CNs but slightly different bond lengths, and therefore EXAFS fitting requires averaging over three scatterers (Supplementary Tables 2 and 3). EXAFS fitting starting from alternative trinuclear models resulted in similar parameters, indicating that the values shown in Table 2 are a true minimum. In addition, full refinement of all parameters including CNs did not result in significant deviation of the parameters as obtained. A *R*-factor of 0.004 indicates a good fit. Naturally, the more complex structure of the trinuclear Cu cluster leads to more first- and second-shell scattering paths compared with the simpler binuclear structure. The quality of the data, in addition to fixing the CNs and amplitudes, allows reliable refinement of all other parameters (number of independent data points is 16, with 13 parameters fitted). The Cu–Cu contributions are significant, and realistic distances and low Debye–Waller factors with low errors are obtained. The Cu-O_EF_ contribution is shifted significantly compared with the model, with large errors, especially in the Debye–Waller factor. This is due to the small contribution of this shell at long distance, and hence this shell cannot be determined with high accuracy. Different four-coordinate Cu clusters, for example, cubane or ring-type structures, were also tested as a starting model for the EXAFS analysis. A full refinement of experimental data with the Cu_4_ model results in exactly the same analysis as presented in the manuscript, which corresponds to the trimeric model rather than a Cu_4_ cubane-type structure. This is another proof that we have refined a true minimum.

### Ultraviolet–visible spectroscopy

Ultraviolet–visible measurements of the Cu-exchanged H-MOR samples were performed with an Avantes Avaspec 2,048 spectrometer in the diffuse reflectance mode. The samples were measured as powders and placed in a quartz flow reactor (6 mm inner diameter) with square optical-grade quartz windows. The reactor was placed horizontally in a lab-made heating chamber with an 8-mm diameter hole on top, through which a high-temperature optical fibre (Avantes FCR-7UV400−2ME-HTX UV-vis reflection probe) could be vertically directed to the reactor. The temperature was measured by a thermocouple located on the bottom of the quartz reactor. In a typical experiment, the UV-vis spectra were collected during treatment in oxygen, nitrogen or methane. The intensity of the diffuse reflectance UV-vis spectra is presented in the form of the Kubelka–Munk function, defined as F(*R*)=(1−*R*)^2^/(2 × *R*) with *R*=*R*_s_/*R*_r_, with *R*_s_—the reflectance of the sample and *R*_r_–the reflectance of the H-MOR parent material used as a reference. The samples were first treated at 450 °C for 1 h in He (flow 16 ml min^−1^), heating at a rate of 10 °C min^−1^. Subsequently, the sample was cooled to ambient temperature, He was replaced with O_2_ and the sample was heated to 450 °C in O_2_ flow (flow 16 ml min^−1^). After cooling of the activated sample to 200 °C, the sample was contacted with CH_4_ (flow 16 ml min^−1^).

## Additional information

**How to cite this article:** Grundner, S. *et al.* Single-site trinuclear copper oxygen clusters in mordenite for selective conversion of methane to methanol. *Nat. Commun.* 6:7546 doi: 10.1038/ncomms8546 (2015).

## Supplementary Material

Supplementary Information Supplementary Figures 1-11, Supplementary Tables 1-5.

## Figures and Tables

**Table 1:** Acidity of Cu-MOR.

**Cu conc. [μmol g**^**−1**^]	**BAS**_**main channel**_† **[μmol g**^**−1**^]	**BAS**_**SP bottom**_‡ **[μmol g**^**−1**^]	**BAS**_**SP pore mouth**_§ **[μmol g**^**−1**^]	**Total BAS [μmol g**^**−1**^]
0	400	310	380	1,090
100	430	270	330	1,030
160	420	270	290	980
290	410	320	160	890
440	440	370	20	830

BAS, Brønsted acid site; MOR, mordenite; SP, side pocket.

Quantification of acid sites for a series of Cu-MOR* (Si/Al=11, Cu/Al≤0.4).

^*^Total concentration of BAS in H-MOR (Si/Al=11) was determined by Na^+^ exchange. For Cu-exchanged MOR, the normalized integral of the O–H vibration of BAS was used for deconvolution and quantification.

^†^Obtained by quantification of the band at 3,612 cm^−1^ (after deconvolution of the band at 3,605 cm^−1^ into 3,612, 3,590 and 3,500 cm^−1^; see Supplementary Fig. 2).

^‡^Calculated by the difference of BAS concentration determined as in * and BAS concentration determined by pyridine.

^§^Calculated by the difference between BAS concentration quantified after *n*-hexane adsorption (band at 3,590 cm^−1^) and BAS concentration in the SP bottom (^‡^); an offset of 70 μmol** **g^−1^ due to dealumination during Cu exchange was substracted for H-MOR.

**Table 2:** Copper EXAFS fitting results.

**Backscatterer**	**Coordination numbers** ***N***^**DFT**^	**Coordination numbers** ***N***^**EXAFS**^	**Distance** ***R***^**DFT**^ **(Å)**	**Distance** ***R***^**EXAFS**^ **(Å)**	**Debye–Waller factor**† **Δ*****σ*****^2^ (Å^2^)**
Cu–O_EF_	2	2.2 (±0.8)	1.80	1.91 (±0.03)	0.003 (±0.003)
Cu–O_F_	1.66	1.6 (±0.5)	2.02	2.04 (±0.07)	0.004 (±0.005)
Cu–O_F_	0.33	0.4 (±0.5)	2.63	2.35 (±0.05)	0.003 (±0.010)
Cu–Cu	0.66	0.7 (±0.4)	2.74	2.86 (±0.04)	0.005 (±0.005)
Cu–Cu	1.33	1.5 (±0.7)	3.04	3.02 (±0.05)	0.010 (±0.006)
Cu–O_EF_	1	1.3 (±1.1)	3.23	3.50 (±0.09)	0.008 (±0.025)

EXAFS, Extended X-ray Absoprtion Fine Structure; DFT, density functional theory; MOR, mordenite.

Comparison of Cu K-edge EXAFS fit results* for O_2_-activated Cu-MOR zeolite with DFT-optimized geometric parameters of [Cu_3_(μ-O)_3_]^2+^ in Cu-MOR.

^*^Combined *k*^1^, *k*^2^ and *k*^3^-weighted fit, 2.4<*k*<12.0 Å, 1<*R*<3.6, *E*_0_=−1 (3), *R*-factor=0.003, *S*_0_^2^ (fixed)=0.9, statistical errors in brackets.

^†^Debye–Waller factors were fixed (to the values obtained in the best fit with set coordination numbers) during EXAFS fit to reduce the number of fitting parameters. The values predicted by DFT calculations are averaged over three Cu scatterers. See also Supplementary Tables 2–5.
