# Supplementary material for: Single-site trinuclear copper oxygen clusters in mordenite for selective conversion of methane to methanol
Source: Nat Commun. 2015 Jun 25;6:7546. doi: 10.1038/ncomms8546 (PMC4491810; doi:10.1038/ncomms8546)
Supplement: Supplementary Information — Supplementary Figures 1-11, Supplementary Tables 1-5. [file ncomms8546-s1.pdf]

Supplementary Information

Supplementary Figures

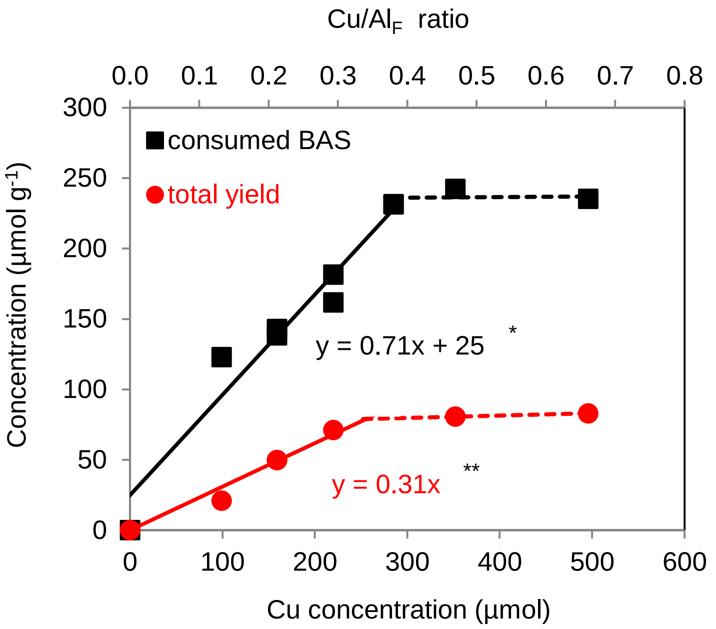

Supplementary Figure 1 | Activity and framework aluminum coordination upon copper loading. Total yield of methane oxidation as a function of Cu concentrate on and the concentration of tetrahedrally coordinated aluminum acting as ion exchange site for Cu^{2+} with total yield for a series of Cu-MOR with Si/Al =21 (b). * The slope of 0.71 indicates an exchange stoichiometry of 2/3 meaning that 2 H^+ are substituted by 3 Cu^{2+}. The offset of 25 µmol/g shows slight dealumination of framework Al (~5%) during Cu-exchange. ** The slope of 0.31 indicates that 3 Cu centers are involved in the oxidation of one methane molecule.

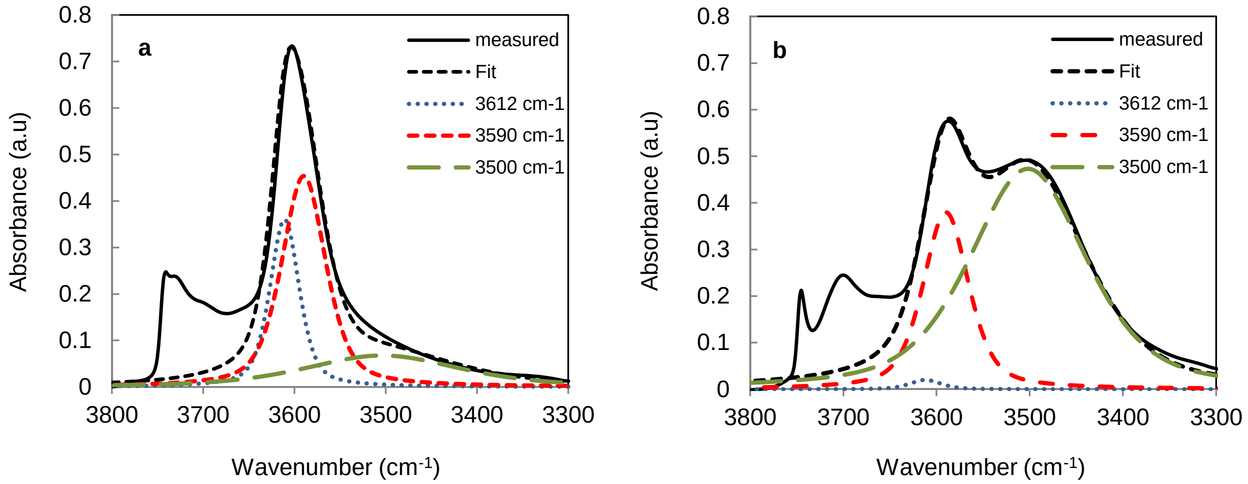

Supplementary Figure 2 | Deconvoluted infrared spectra of H-MOR. Infrared spectra of the OH stretching vibrations of the BAS of H-MOR after activation at 723 K (**a**) and during adsorption of n-hexane at 303 K (**b**). BAS are deconvoluted in bands attributed to main channel OH (3,612 cm^{-1}), side pocket OH stretching vibrations (3,590 cm^{-1}) and perturbed O-H stretching vibrations (3,500 cm^{-1}).

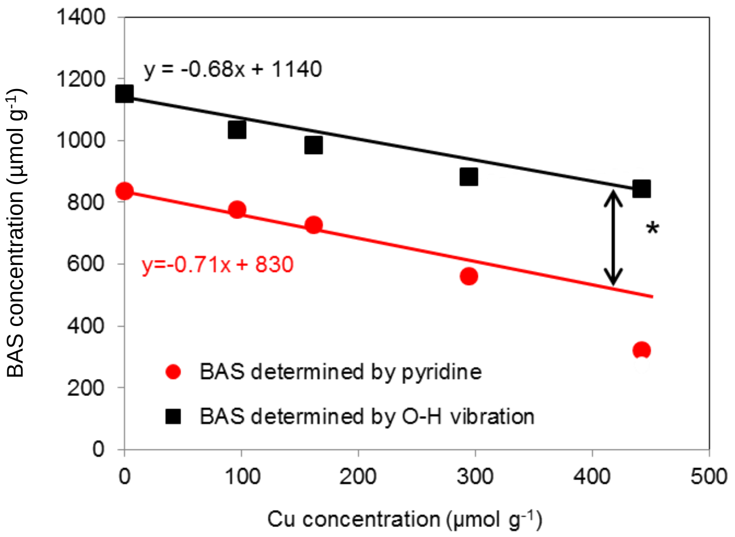

Supplementary Figure 3 | Infrared spectroscopic quantification of Brønsted acid sites in Cu-MOR. Plot of BAS concentration quantified by OH stretching vibrations (3,605 cm^{-1}) of the BAS of H-MOR after activation at 723 K and quantified by vibrational band of the pyridinium ion after adsorption of pyridine (1,545 cm^{-1}). * The gap between the BAS concentration quantified by OH stretching intensity and pyridine adsorption is due to BAS in the bottom of the side pockets which are inaccessible to pyridine. The slopes of -0.68 and -0.71 respectively indicate that 2 H^+ are exchanged for 3 Cu^{2+} in the pore mouth of the side pockets, which are accessible for pyridine.

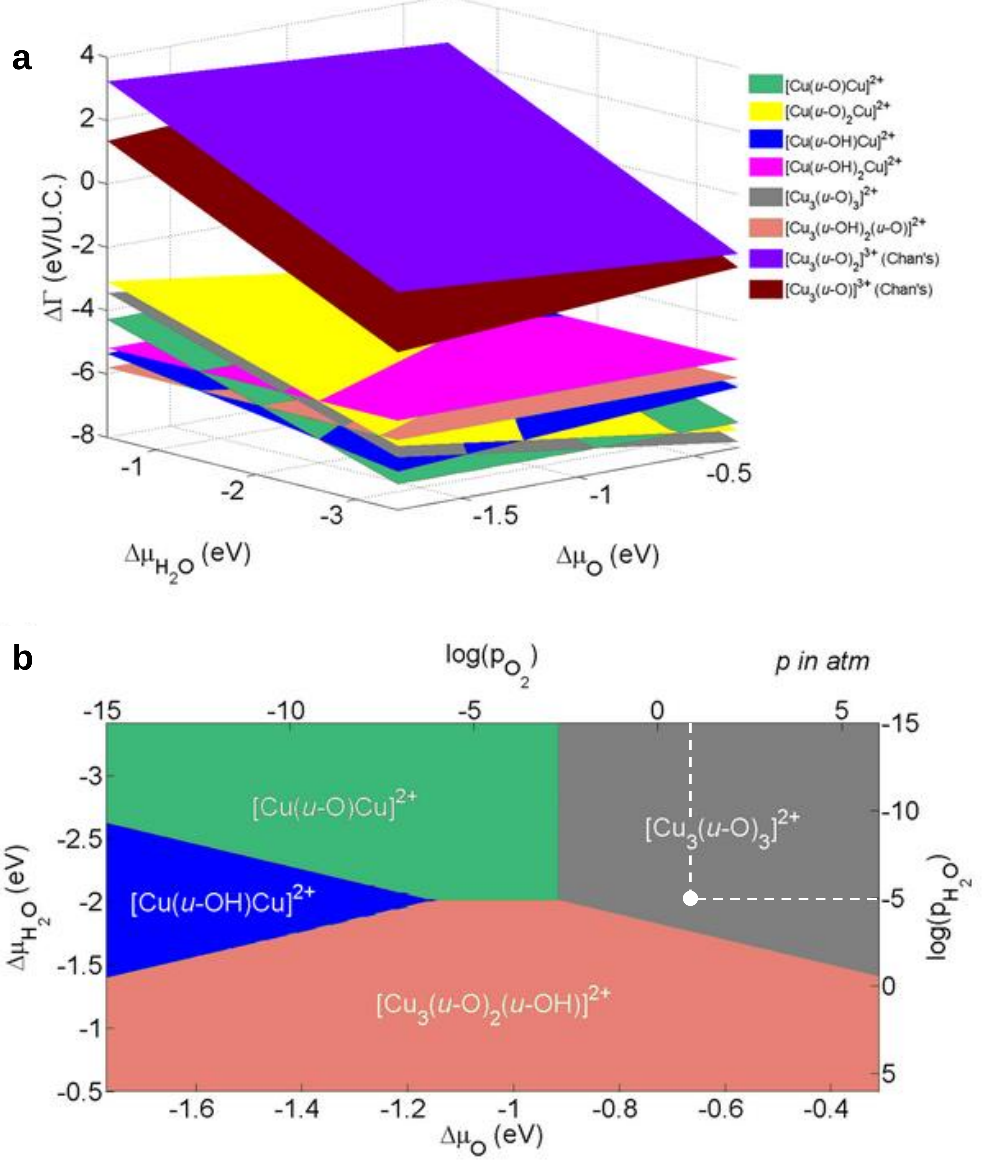

Supplementary Figure 4 | Ab initio thermodynamic analysis. Reaction free energy as a function of water and oxygen chemical potential (**a**) and 2D projection of the lowest free energy $Cu_xO_mH_n$ species in MOR supercell ($\Delta\Gamma$ (eV/U.C.), cf. equation. (1)) as a function of oxygen chemical potential ($\Delta\mu_O$) and water chemical potential ($\Delta\mu_{H_2O}$). $\Delta\mu_O$ and $\Delta\mu_{H_2O}$ are translated into pressure scales at T = 700 K (**b**); The conditions of our experiments (1 atm O_2, 10 ppm H_2O) and consequently the location of the activated Cu-MOR are marked as white dot.

$$\frac{2m-2x-n+4}{4}O_2 + \frac{n-4}{2}H_2O + x(CuO) + 4H - MOR \leftrightharpoons Cu_xO_mH_n/MOR \quad (1)$$

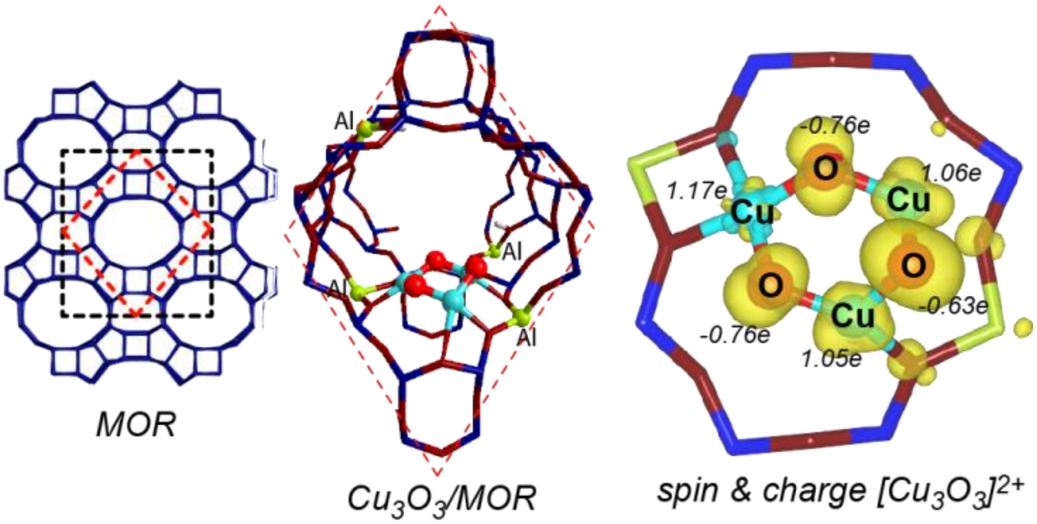

Supplementary Figure 5 | Structure, location and electronic properties of $[Cu_3(\mu\text{-}O)_3]^{2+}$ cluster in MOR zeolite model predicted by periodic DFT calculations (Si/Al=11). The right panel depicts spin-density and calculated atomic Bader charges for the extraframework Cu cluster.

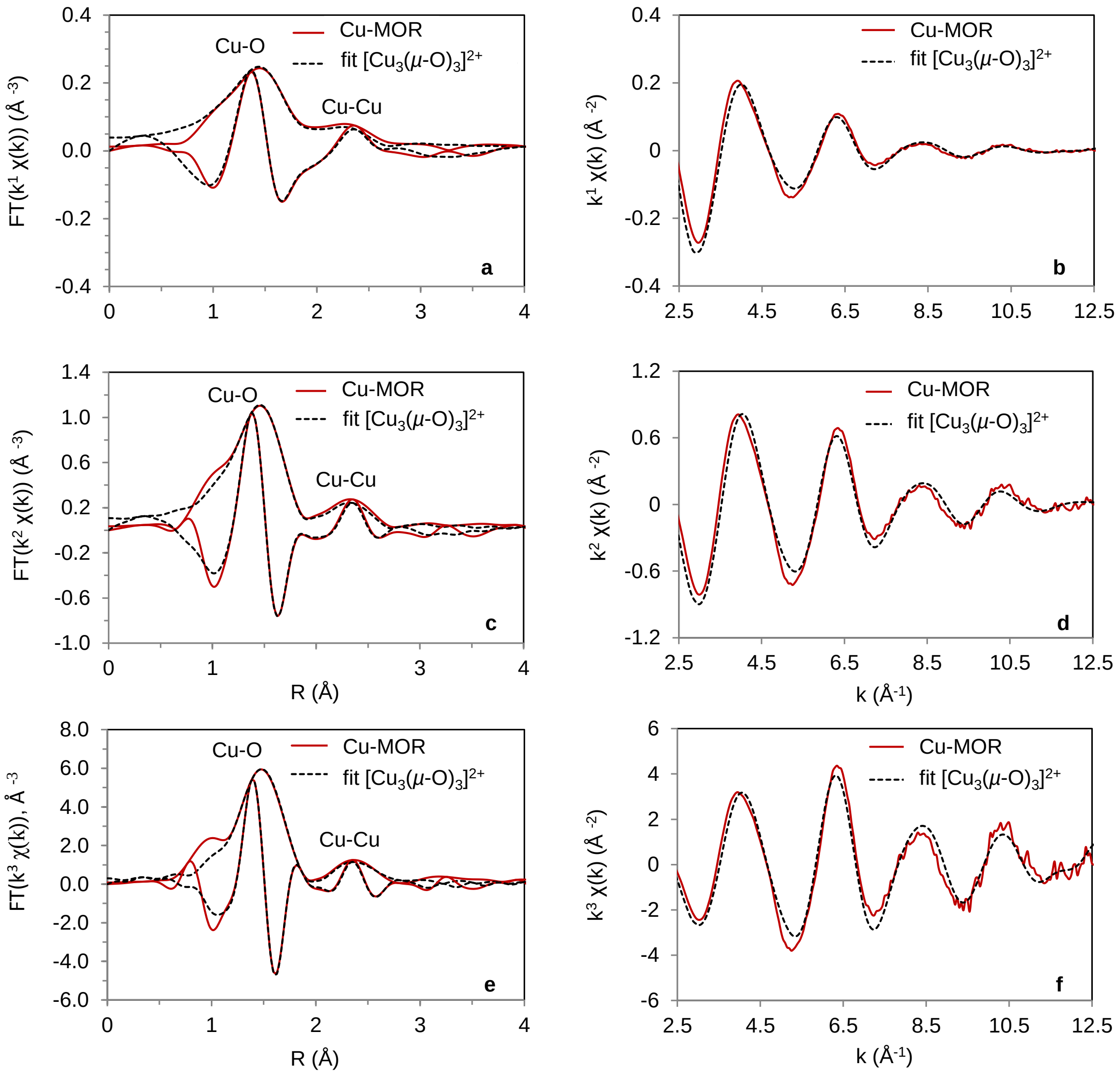

Supplementary Figure 6 | Copper EXAFS data and fitting for Cu-MOR. Comparison of the k^1,k^2 and k^3-weighted Fourier transformed EXAFS (**a,c,e**) at the Cu K-edge of the Cu-MOR zeolite activated in O_2 at 723 K and EXAFS simulation of an intrazeolite trinuclear $[Cu_3(\mu\text{-}O)_3]^{2+}$ complexes and (**b,d,f**) the corresponding k^1,k^2 and k^3-weighted experimental EXAFS oscillations and their simulation using the DFT computed model. Color key: measured spectra (red lines), simulated spectra (black dotted lines).

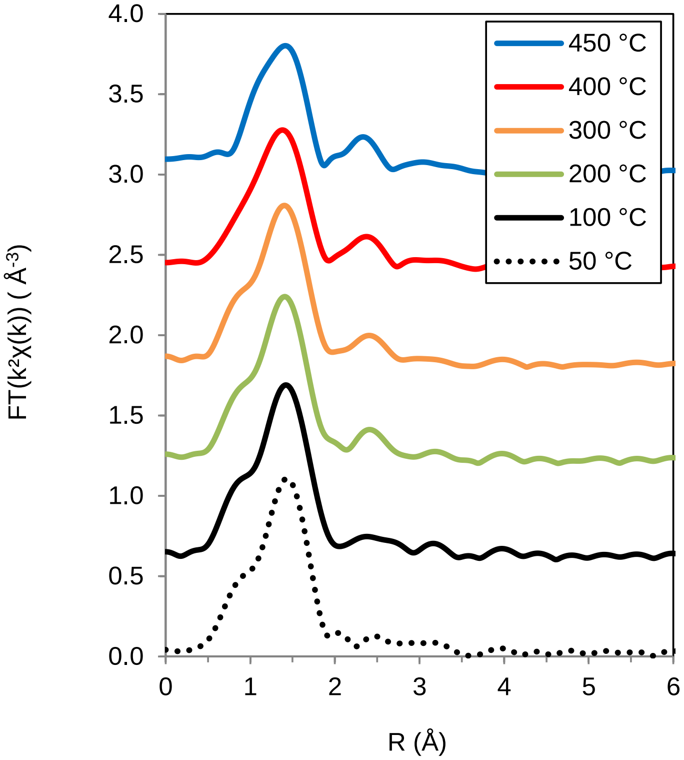

Supplementary Figure 7 | In situ Copper EXAFS of Cu-MOR. FT EXAFS of Cu-MOR sample during activation in pure O_2 flow with a heating rate of 10 K/min.

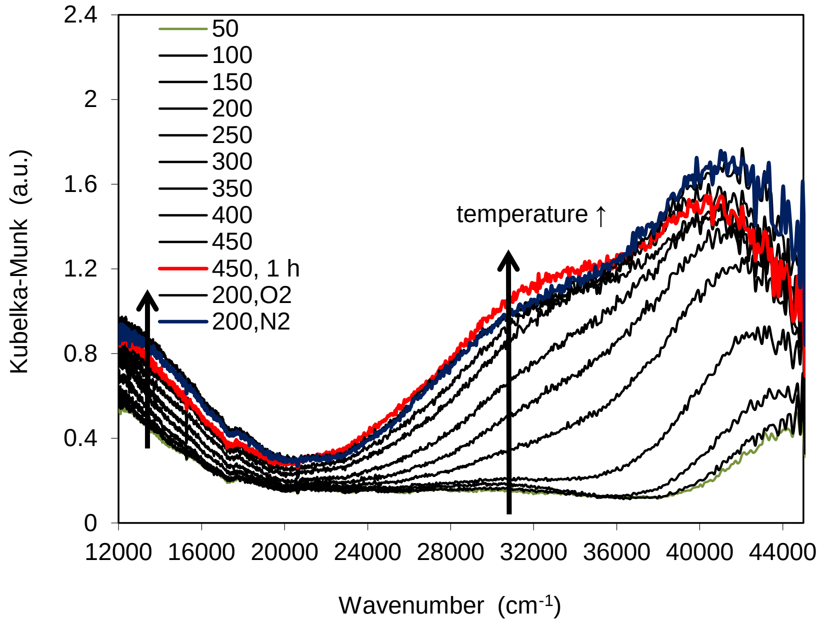

Supplementary Figure 8 | In situ UV-vis spectra of Cu-MOR during activation. UV-vis spectra of fresh Cu-MOR during activation in O_2 up to 723 K. After treatment, temperature was decreased to 473 K under O_2 and then flushed in N_2 for 1 hour at 473 K (final spectra in blue).

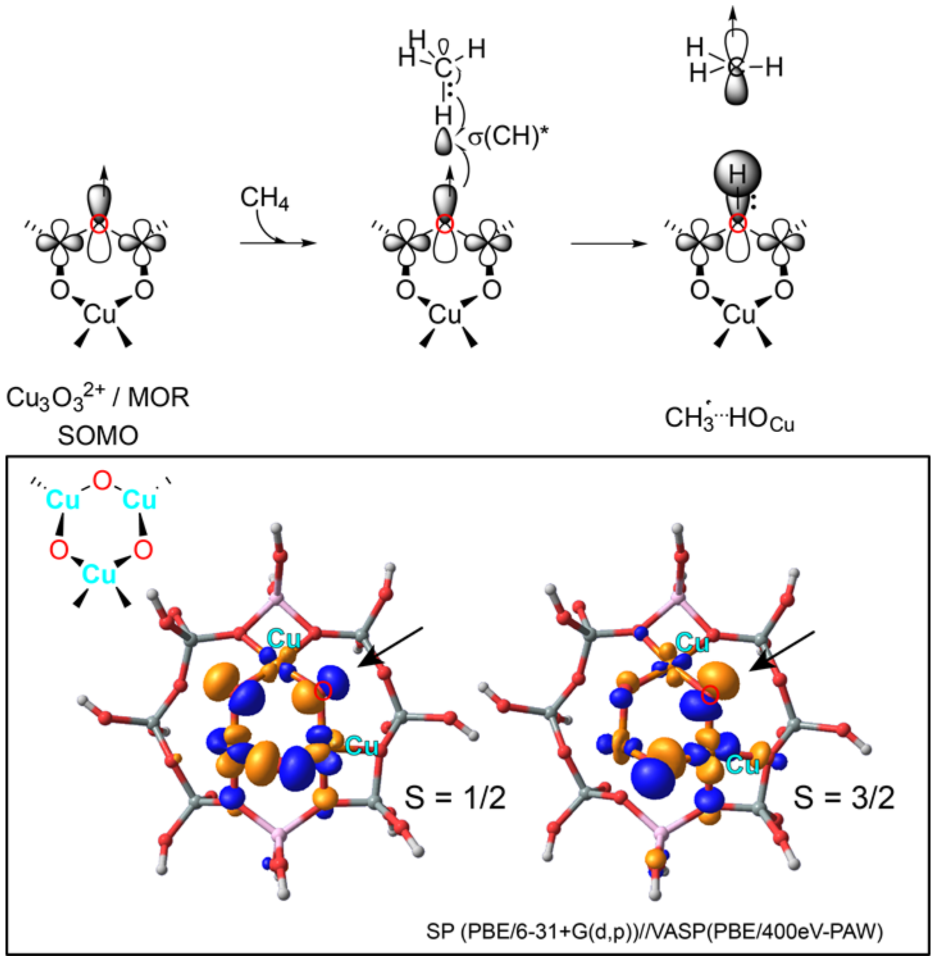

Supplementary Figure 9 | Molecular orbital analysis A schematic representation of orbital interactions involved in C-H bond activation in methane by the reactive O_{EF} in the trinuclear $[Cu_3O_3]^{2+}$ cluster. The insert depicts the DFT-computed SOMO of the cluster model (Gaussian 09, PBE/6-31+G(d,p)) cut from the periodic DFT-optimized Cu_3O_3/MOR structure in S=1/2 and S=3/2 spin states (the reactive O moiety in the cluster is shown with the arrow).

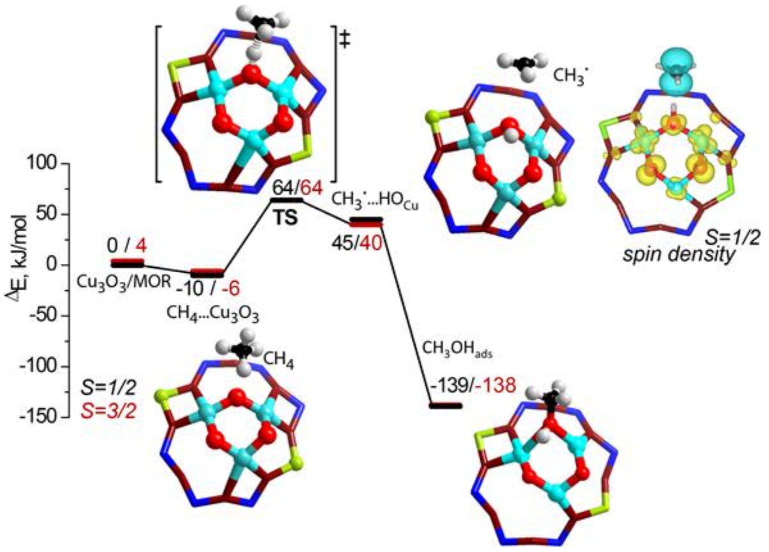

Supplementary Figure 10 | Reaction energy profiles DFT-computed reaction energy profiles (ground spin states of S=1/2 and S=3/2) for methane activation by the trinuclear $[Cu_3(\mu\text{-}O)_3]^{2+}$ in MOR zeolite. The spin density of $CH_3^{\bullet}\cdots HO_{Cu}$ is inserted.

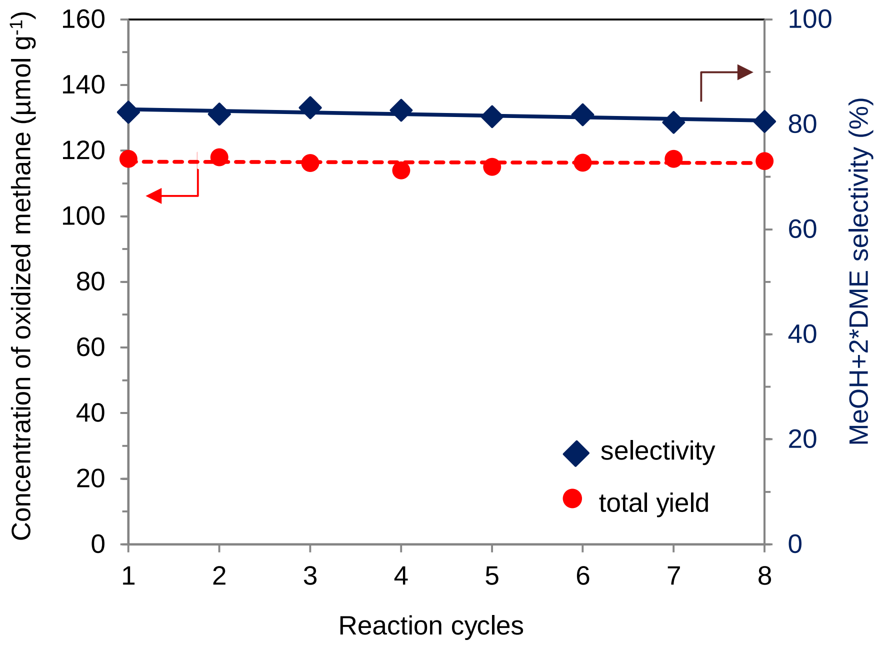

Supplementary Figure 11 | Catalytic stability of Cu-MOR. Stability test of Cu-MOR for selective partial oxidation of methane to methanol. Recycling of the catalyst over 8 cycles with activation at 450 °C for 1h, methane loading at 200 °C for 30 min and steam treatment at 135 °C for 30 min in each cycle.

Supplementary Tables

Supplementary Table 1 | BET analysis of Cu-exchanged mordenites.

Sample	Si/Al ratio	Cu loading [wt.%]	BET surface area [m^2 g^{-1}]	$V_{micropore}$ [cm^3 g^{-1}]
H-MOR	11	0	534	0.20
Cu-MOR	11	1.0	499	0.18
Cu-MOR	11	2.9	501	0.19
Cu-MOR	21	1.0	517	0.17

Supplementary Table 2 | Calculated scattering paths based on DFT optimized model of Cu-MOR. DFT optimized geometric parameters of $[Cu_3(\mu\text{-}O)_3]^{2+}$ in Cu-MOR predicted by DFT calculation. All paths in a distance ≤ 3.5 Å were taken into account.

Scatterer	Backscatterer	Number of paths (DFT)	Distance [Å]
Cu1	O	1	1.79
	O	1	1.80
	O	1	1.96
	O	1	2.02
	Cu	1	3.04
	Cu	1	3.05
Cu2	O	1	1.77
	O	1	1.78
	O	1	2.04
	O	1	2.11
	Cu	1	2.74
	Cu	1	3.05
	O	1	3.23
	O	1	3.41
	O	1	3.44
Cu3	O	1	1.77
	O	1	1.82
	O	1	1.99
	O	1	2.63
	Cu	1	2.74
	Cu	1	3.04
	O	1	3.20
	O	1	3.44

Supplementary Table 3 | Average coordination number for copper in Cu-MOR. Determination of average coordination numbers per copper atom for the DFT model of $[Cu_3(\mu\text{-}O)_3]^{2+}$ in Cu-MOR.

Path	Average Distance[a] R^{DFT} [Å]	Number of paths (DFT)[b]	Coordination number N[c]
Cu-O_{EF}	1.80 (±0.03)	6	2
Cu-O_F	2.01 (±0.05)	5	1.66
Cu-O_F	2.63 (±0.01)	1	0.33
Cu-Cu	2.74 (±0.01)	2	0.66
Cu-Cu	3.04 (±0.01)	4	1.33
Cu-O_{EF}	3.23 (±0.03)	2	0.66

[a]Cu-O and Cu-Cu paths averaged values derived from data in Supplementary Table 2 (values predicted by DFT calculations, averaged over 3 Cu scatterers, statistical errors in brackets); [b]Number of paths that were averaged; [c]Coordination numbers averaged over 3 copper atoms obtained by dividing the number of paths in b by the number of Cu atoms of the cluster.

Supplementary Table 4| EXFAS fitting of Cu-MOR with dimeric copper structures. Comparison of Cu K-edge EXAFS fit results[a] for O_2-activated Cu-MOR zeolite with DFT optimized geometric parameters of $[Cu_2(\mu\text{-}O)]^{2+}$

Backscatterer	Coordination number N[b]	Distance R^{DFT} [Å]	Distance R^{EXAFS} [Å]	Debye-Waller factor $\Delta\sigma^2$ [Å²]
Cu-O_{EF}	3	1.95	1.94 (±0.04)	0.005 (±0.001)
Cu-Cu	1	2.94	2.92 (±0.07)	0.012 (±0.004)
Cu-O_F	1	3.56	3.52 (±0.04)	0.011 (±0.018)

[a]Combined k^1, k^2 and k^3-weighted fit, 2.4<k<12.0, 1<R<3.6, E_0= -2 (2), R factor= 0.009, S_0^2(fixed)= 0.9, statistical errors in brackets; [b]Coordination numbers were fixed during EXAFS fit to reduce the number of fitting parameters (values predicted by DFT calculations).

Supplementary Table 5 | EXFAS fitting of Cu-MOR with dimeric copper structures. Comparison of Cu K-edge EXAFS fit results[a] for O_2-activated Cu-MOR zeolite with DFT computed geometrical parameters of $[Cu_2(\mu\text{-}O)_2]^{2+}$

Backscatterer	Coordination number N[b]	Distance R^{DFT} [Å]	Distance R^{EXAFS} [Å]	Debye-Waller factor Δσ² [Å²]
$Cu\text{-}O_{F/EF}$	4	1.95	1.94 (±0.05)	0.007 (±0.005)
Cu-Cu	1	2.94	2.91 (±0.07)	0.012 (±0.003)
$Cu\text{-}O_F$	1	3.56	3.46 (±0.04)	0.008 (±0.012)

[a]Combined k^1, k^2 and k^3-weighted fit, 2.4<k<12.0, 1<R<3.6, E_0= -2 (2), R factor= 0.008, S_0^2(fixed)= 0.9, statistical errors in brackets; [b]Coordination numbers were fixed during EXAFS fit to reduce the number of fitting parameters (values predicted by DFT calculations).
